# Ubiquitin ligase CHAF1B induces cisplatin resistance in lung adenocarcinoma by promoting NCOR2 degradation

**DOI:** 10.1186/s12935-020-01263-2

**Published:** 2020-05-25

**Authors:** Lian Gong, Yi Hu, Dong He, Yuxing Zhu, Liang Xiang, Mengqing Xiao, Ying Bao, Xiaoming Liu, Qinghai Zeng, Jianye Liu, Ming Zhou, Yanhong Zhou, Yaxin Cheng, Yeyu Zhang, Liping Deng, Rongrong Zhu, Hua Lan, Ke Cao

**Affiliations:** 1 Department of Oncology, Third Xiangya Hospital of Central South University, Changsha, 410013 China; 2 Department of Respiratory, The Second People’s Hospital of Hunan Province, Changsha, 410007 China; 3 Department of Gastroenterology, Third Xiangya Hospital of Central South University, Changsha, 410013 China; 4 Department of Dermatology, Third Xiangya Hospital of Central South University, Changsha, 410013 China; 5 Department of Urology, Third Xiangya Hospital of Central South University, Changsha, 410013 China; 6 Cancer Research Institute and Key Laboratory of Carcinogenesis of the Chinese Ministry of Health, Central South University, Changsha, 410078 China; 7 Department of Gynaecology, Third Xiangya Hospital of Central South University, Changsha, 410013 China

**Keywords:** Lung adenocarcinoma, Cisplatin, Drug sensitivity, Ubiquitin ligase, CHAF1B, NCOR2

## Abstract

**Background:**

Lung cancer is the most common malignant tumor in the world. The Whole-proteome microarray showed that ubiquitin ligase chromatin assembly factor 1 subunit B (CHAF1B) expression in A549/DDP cells is higher than in A549 cells. Our study explored the molecular mechanism of CHAF1B affecting cisplatin resistance in lung adenocarcinoma (LUAD).

**Methods:**

Proteome microarray quantify the differentially expressed proteins between LUAD cell line A549 and its cisplatin-resistant strain A549/DDP. Quantitative real-time quantitative polymerase chain reaction (qRT-PCR) and Western blot (WB) confirmed the CHAF1B expression. Public databases analyzed the prognosis of LUAD patients with varied LUAD expression followed by the substrates prediction of CHAF1B. Public databases showed that nuclear receptor corepressor 2 (NCOR2) may be substrates of CHAF1B. WB detected that CHAF1B expression affected the expression of NCOR2. Cell and animal experiments and clinical data detected function and integrating mechanism of CHAF1B compounds.

**Results:**

Proteome chips results indicated that CHAF1B, PPP1R13L, and CDC20 was higher than A549 in A549/DDP. Public databases showed that high expression of CHAF1B, PPP1R13L, and CDC20 was negatively correlated with prognosis in LUAD patients. PCR and WB results indicated higher CHAF1B expression in A549/DDP cells than that in A549 cells. NCOR2 and PPP5C were confirmed to be substrates of CHAF1B. CHAF1B knockdown significantly increased the sensitivity of cisplatin in A549/DDP cells and the upregulated NCOR2 expression. CHAF1B and NCOR2 are interacting proteins and the position of interaction between CHAF1B and NCOR2 was mainly in the nucleus. CHAF1B promotes ubiquitination degradation of NCOR2. Cells and animal experiments showed that under the action of cisplatin, after knockdown of CHAF1B and NCOR2 in A549/DDP group compared with CHAF1B knockdown alone, the cell proliferation and migratory ability increased and apoptotic rate decreased, and the growth rate and size of transplanted tumor increased significantly. Immunohistochemistry suggested that Ki-67 increased, while apoptosis-related indicators caspase-3 decreased significantly. Clinical data showed that patients with high expression of CHAF1B are more susceptible to cisplatin resistance.

**Conclusion:**

Ubiquitin ligase CAHF1B can induce cisplatin resistance in LUAD by promoting the ubiquitination degradation of NCOR2.

## Background

Lung cancer is the most common malignant tumor in the world, and its morbidity and mortality rank the first in the world and China [1, 2]. Because the early symptoms of lung cancer patients are not obvious, the cancer was in advanced stage when it was discovered, chemotherapy plays an important role in advanced lung cancer. The first-line chemotherapy for lung cancer is based on platinum-based chemotherapy, and cisplatin is the most commonly used platinum drugs. But in general, patients with lung cancer are resistant to cisplatin in treatment, which leads to the failure of treatment. Lung adenocarcinoma accounts for 40–55% of lung cancer. Therefore, it is very important to explore the specific molecular mechanism of cisplatin resistance in lung adenocarcinoma and provide a new target of chemotherapy sensitization for lung adenocarcinoma patients.

The mechanism of cisplatin resistance in lung cancer is complicated. So far, the mechanism of cisplatin resistance is closely related to the accumulation of platinum drugs in the cells, DNA damage repair [3], apoptosis, epithelial-mesenchymal transition, tumor stem cell characteristics [4] and so on [5–7]. The proteins in above signaling pathways are usually regulated by various factors such as genomics, epigenetics and post-translational modification of proteins. Recent studies have shown that cisplatin resistance is closely related to post-translational modification of proteins [8, 9], which directly affects the spatial conformation, activity and stability of the protein at the protein level, and then regulates various functions of the protein to regulate the life activities. There are more than 400 types of post-translational modifications, including phosphorylation, acetylation, ubiquitination, methylation, and glycosylation. Recent studies have shown that cisplatin resistance is closely related to protein ubiquitination [10].

Protein ubiquitination refers to the process in which ubiquitin (a highly conserved short peptide consisting of 76 amino acids, widely expressed in eukaryotes) is linked to a target protein by a series of enzymes to modify the target protein specifically. E3s play an important role in recognizing substrates during ubiquitination. Ubiquitin ligase MKRN1 regulates translating ribosome by recognizing, binding and promoting ubiquitination of its substrate PABPC1 [11]. Ubiquitin ligase Speckle-type BTB-POZ protein (SPOP) limits inflammation by promoting ubiquitination of the innate signal transducer, myeloid differentiation primary response protein 88 (MYD88) [12].During mitosis induction, ubiquitin ligase CUL4-RING (CRL4s) raises the direct substrate WIPI2/ATG18B (WD repeat domain, phosphoinositide interacting 2), an autophagy-related (ATG) protein that plays a critical role in autophagosome biogenesis through DDB1 (damage specific DNA binding protein 1), thus leading to the polyubiquitination and proteasome degradation of WIPI2 and inhibiting autophagy [13]. It has been proved that many of ubiquitin ligases are closely related to tumor development, malignant phenotype, and cisplatin resistance in cancer [14]. For example, Ubiquitin ligase HOIP [15] inhibits apoptosis induced by cisplatin. Ubiquitin ligase hakai [16] regulates the growth and invasion of non-small cell lung cancer cells and increases their sensitivity to cisplatin.

We conducted a proteomic microarray to identify a series of significant changes protein between lung adenocarcinoma cell line A549 and lung adenocarcinoma cisplatin-resistant strain A549/DDP to explore the reason of cisplatin resistance in patients with lung adenocarcinoma. Compared with A549, the activity of ubiquitin ligases in A549/DDP were significantly increased. Therefore, our study explored whether ubiquitin ligase regulated cisplatin sensitivity in lung adenocarcinoma and its regulatory mechanism. The research may provide scientific bases and new ideas for the reversal of cisplatin resistance and individualization in clinical lung adenocarcinoma.

## Methods

### Cell culture and transfection

A549 and A549/DDP cell lines were purchased from Central South University-Changsha in China and cultured in RPMI-1640 cell culture medium (Sigma-Aldrich, St Louis, MO, USA) supplemented with 10% foetal bovine serum (FBS), 100 units/ml penicillin and 100 μg/ml streptomycin. The culture medium of A549/DDP was added with 2 μg/ml of DDP and placed in the incubator of 5% CO2 and 95% air in 37 °C for culture. All RNA inhibitors and negative control si-RNA are from GenePharma company (Shanghai, China). The sequences for si-RNA were as follows: si-CHAF1B [17] 5′-ACGGAAAGUCUGGACCCUUTT-3′; si-CDC20 [18] 5′-CGAAAUGACUAUUACCUGA-3′; si-PPP1R13L [19] 5′-GCATCACTGCCTTGCACAA-3′; si-NCOR2 5′-GAAAGGCACUCAUGGGUAATT-3′; Negative control 5′-UUCUCCGAACGUGUCACGUTT-3′. Then A549 and A549/DDP cells were transfected by siRNA using Lipofectamine^TM^ 2000 (Invitrogen Life Technologies) according to the manufacturer’s recommended method.

### Quantitative real-time polymerase chain reaction (qRT‐PCR)

TRIzol Reagent (Invitrogen) was used to extract total RNA from A549 and A549/DDP cells. Next, reverse transcription was carried out using the RevertAid First Strand cDNA Synthesis Kit (Thermo Fisher Scientific, Waltham, MA, USA) to cDNA. Real-time PCR was performed using SYBR Green Premix PCR Master Mix (Roche, Mannheim, Germany). The relative quantification of CHAF1B, CDC20, PPP1R13L, and TRIP12 was calculated according to the ΔΔCT method. The primer sequences for amplification of CHAF1B were as follows: forward: 5′-TCATACCAAAGCCGTCAA-3′; reverse: 5′-GCCCAGCAAATATCATACAC-3′. The primer sequences for CDC20 were as follows: forward: 5′-GGCAAATCCAGTTCCAAG-3′; reverse: 5′-TTCAGTCTGTTCTGATAACCCT-3′. The primer sequences for TRIP12 were as follows: forward: 5′-GAAGACAGCGATGACGAT-3′; reverse: 5′-ATGTTATACGGCAGCAAA-3′. The primer sequences for PPP1R13L were as follows: forward: 5′-TGGAGGCTGGCGATGTGGA-3′; reverse: 5′-GCTGGTACTGTTTCTTGTGGGT-3′.

### Western blot analysis

The protein in A549 and A549/DDP and tissues was extracted by Radioimmunoprecipitation assay buffer (RIPA) (Auragene, Changsha, China). Proteins were quantified by bicinchoninic acid (BCA) protein assay kit (Thermo Scientific). Proteins were separated on a 10% SDS-PAGE gel and transferred onto nitrocellulose membranes, and then incubated with 5% BSA for 2 h at 25 °C. The membranes were incubated with Anti-CHAF1B antibodies (1:500; ab72520, Abcam); Anti-NCOR2 antibodies (1:500; ab24551, Abcam); Anti-PPP5C antibodies (1:500; sc271612, Santa Cruz) was incubated overnight at 4 °C. Then it was incubated with a secondary HRP-labelled goat anti-rabbit IgG antibody (1:4000; Abcam) for 1 h at room temperature. Immunoassay were performed by ECL combined with Western Blot system (Auragene, Changsha, China). GAPDH (1:4000; ab125247, Abcam) was used as the internal control of Western blotting.

### Methyl‐thiazolyl‐tetrazolium assay

The cells were cultured to logarithmic growth stage. After digestion, the cells were inoculated into a 96-well plate at a density of 1 × 10^4^/ml. The IC25 concentration of DDP in A549/DDP was set according to the experimental design. After drug treatment, the cells were incubated with suitable growth conditions. After 24, 48 and 72 h, 50 μl 1*MTT (Sigma-Aldrich, St Louis, MO, USA) was added into each well. Then the plates were cultured for 4 h. 150 μl DMSO was added to each well to dissolve purple formazan crystals. The microplate reader (Bio-Rad, USA) was employed to measure the optical density (OD) of each well at 490 nm. The experiment was repeated in triplicate.

### Flow cytometry assay

After transfection and DDP treatment, cell apoptosis was determined following protocol of Annexin V-(FITC)/PI apoptosis Detection Kit (KeyGEN Biotech, Nanjing, China). Cell apoptotic status was analysed by flow cytometry (FACSCanto II, BD).

### Co-Immunoprecipitation and ubiquitination assay

After transfection, the cells were lysed with RIPA lysate. Anti-NCOR2 antibodies (1:500; ab24551, Abcam) and human IgG (Bioss, bs-0297P) were added as precipitation antibodies for one night at 4 °C. Add 20 μl fully resuspended Protein A + G Agarose for 2 h at 4 °C. Centrifuge for 5 min at 2500 rpm, then remove the supernatant. Co-IP samples was isolated by SDS–PAGE, immunoblotted with anti-ubiquitin antibody, and analyzed by Western blot.

### Immunofluorescence staining and confocal analysis

When the A549/DDP cells were grown until the cell confluence reached approximately 70%, the fixation and staining treatment are started. After that, added primary antibody overnight. The first antibody was anti-CHAF1B antibody (1:200, ab72520 Abcam) and NCOR2 (1:100, ab24551abcam). The cells were then incubated with Alexa Fluor^®^ 488 Goat Anti-Mouse antibody labeled diluted 1:100 in blocking solution. Nuclei were stained with DAPI for 5 min. Cells were examined with LeicaTCS-SP5 laser confocal scanning microscope.

### Wound healing assay

After transfection and treatment with DDP for 24 h, the experiment was carried out in a 24-well plate. Add about 1 × 10^5^ cells/ml cells to each well. Culture cells until the bottom of the hole is covered. Use a sterile 20 μl pipette tip to draw a line in the middle of the cell and take a picture of it as a 0 h control. The distance to the open area was measured and photographed at 48 h after the scratch.

### Colony formation assay

Cells after being transfection were cultured to logarithmic growth phase and planted into a 6 cm culture dish. Cisplatin was added to the wells according to the experimental design. After 14 days of culture, cells were fixed in 4% paraformaldehyde for 15 min and stained by 0.1% crystal violet for 30 min. Then the cell colony formation was photographed and the clone was counted if the cell number of the clone totaled 50.

### Animal experiments

Female BALB/C nude mice (Purchased from Hunan SJA Laboratory Animal Co., Ltd) aged 4–6 weeks were used to establish a subcutaneously implanted tumour model. A549/DDP cells were transfected with si-RNA according in accordance with the experimental design. After transfection, 1 × 10^6^ cells were injected subcutaneously into mice. Cisplatin was administered when the tumors could be touched. The size of the tumors was monitored and calculated (a: longest diameter, b: shortest diameter, once every 3 days, six times in a row) with the following formula: V (tumour volume: mm^3^) = a * b^2^ * 0.52. 25 days later, the mice were killed, and the weights of the xenograft tumour tissues were measured. These tumour tissues were then fixed for further histological analysis. All animal experiments were approved by Central South University of China and carried out in accordance with international guidelines and programs.

### Clinical tissues

21 paraffin-embedded lung adenocarcinoma specimens were obtained from the Third Xiangya Hospital of Central South University (Changsha, China) from 2016 to 2018. The inclusion criteria were as follows: (1) Histopathological examination confirmed lung adenocarcinoma; (2) No operation or recurrence after operation, with assessable lesions. Patients with advanced lung cancer who have lost the opportunity of surgery, with postoperative recurrence of lung cancer who have assessable lesions, or patients who are intolerant or unwilling to undergo surgical treatment for other reasons were included in the study; and (3) Patients received chemotherapy with cisplatin, but no indication of using molecular targeted drugs and immunotherapy drugs. The World Health Organization (WHO) criteria and the United States Joint Commission on Cancer (AJCC) criteria (8th edition of the Tumor Node Metastasis (TNM) classification were used to classify and stage tumors. The sensitivity or resistance of 21 patients to cisplatin were analyzed by computed tomography (CT) before and after cisplatin treatment. The 21 patients were divided into two groups: the cisplatin sensitive group (n = 10) and the cisplatin resistant group (n = 11). The responses to chemotherapy were scored using a tumor regression grade (TRG) developed by the American Joint Commission on Cancer and the College of American Pathology. We divided the patients with a TRG of 0 or 1 into the cisplatin sensitive group and those with TRG 2 or 3 into the cisplatin sensitive group. These clinical materials were used for research purposes with the prior consent of the patient and approval from the Research Ethics Committee of the Third Xiangya Hospital.

### Immunohistochemistry

Tissue derived from animal experiments and clinical specimens. Firstly, sections were prepared, dewaxed, hydrated. Then, paraffin sections were repaired with tissue antigen. The sections were treated with 50 μl peroxidase to block the endogenous peroxidase activity for 10 min. Subsequently, the sections were probed with polyclonal anti‐Ki-67 (1:100; GTX16667, genetex), anti‐Active-Caspase 3 (1:200; ab2302, abcom) and CHAF1B (1:500; ab72520, Abcam) at 4 °C overnight. The bound antibodies were detected with a biotin-labeled second antibody, diaminobenzidine was added for visualizing, and hematoxylin was used as a counterstain. Finally, the sections were dehydrated and cleared and sealed with neutral gum. Two pathologists who were blinded without knowledge of the clinical or histopathological data scored the samples. The score was determined by the proportion of positive tumor cells and the degree staining intensity [20].

### Statistical analysis

The data were statistically analyzed with GraphPad Prism (version 7.0; GraphPad Software, San Diego, CA, USA). All results were expressed by mean ± standard deviation (SD) from 3 independent experiments. A comparison between the two samples was measured by Student *t* test (e.g., qRT-PCR data). Multiple comparisons were performed using a Bonferroni’s test and Tukey’s test (e.g., flow cytometry, wound healing assay, colony formation assay, and MTT assay). statistically significant was considered when the p value was < 0.05.

## Results

### The ubiquitin ligase CHAF1B in the whole proteome of A549/DDP cell line is significantly up-regulated and can regulate the sensitivity of lung adenocarcinoma to cisplatin

**Fig. 1:**
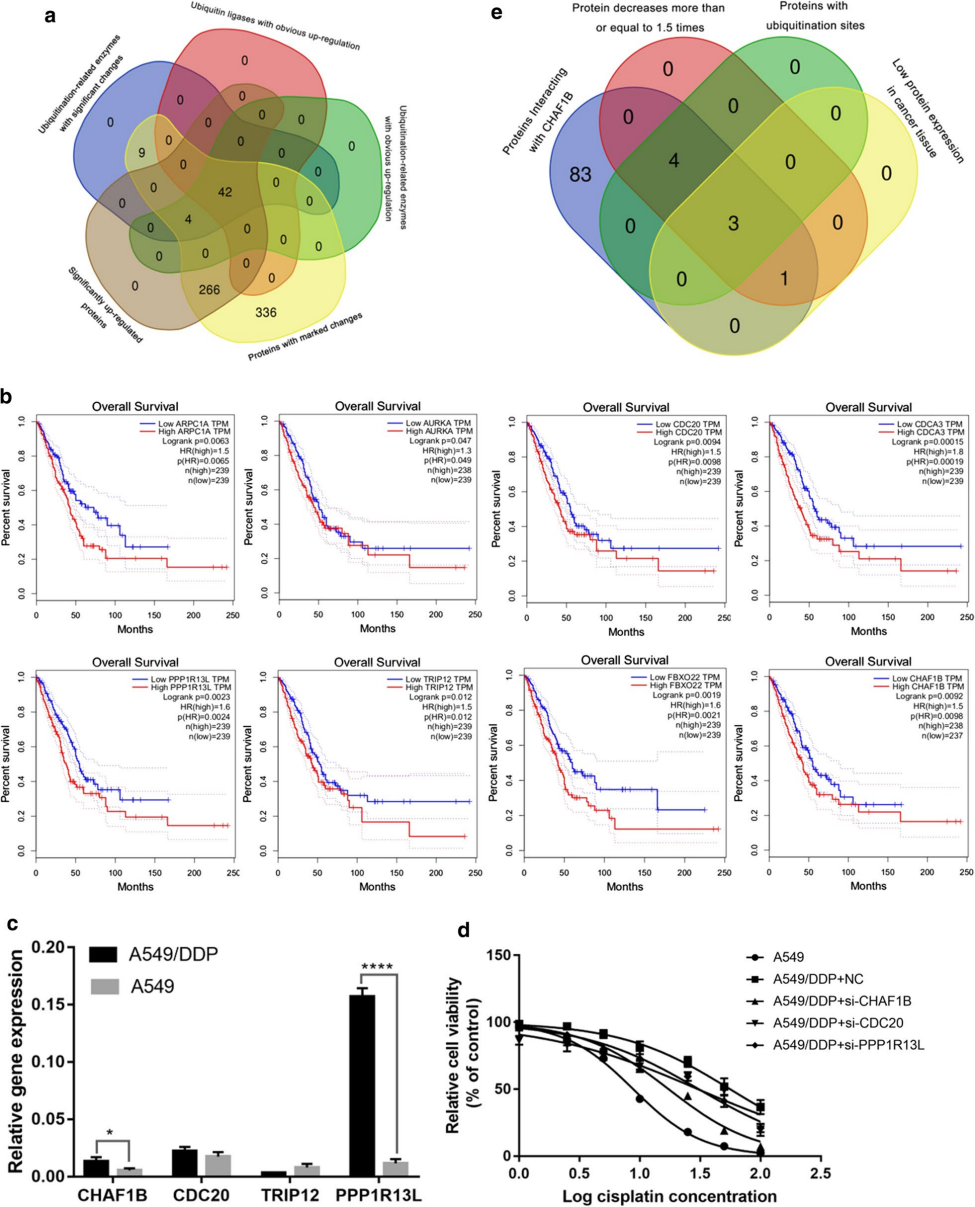
High CHAF1B expression was negatively correlated with prognosis of LUAD patients and regulated cisplatin sensitivity. **a** Whole proteome chips showed chat there are 657 significantly changed protein, of which 312 significantly up-regulated protein, and 345 significantly down-regulated protein. There are 46 significantly up-regulated ubiquitination enzymes, including 42 E3; **b** According to the public database, 8 E3 high expressions, including: ARPC1A, AURKA, CDC20, CDCA3, CHAF1B, FBXO22, PPP1R13L, TRIP12, were negatively correlated with the prognosis of patients with lung adenocarcinoma; **c** PCR results indicated that the expression of CHAF1B, PPP1R13L and CDC20 in A549/DDP was significantly higher than that in A549; d After knocking down CHAF1B, PPP1R13L and CDC20, the proliferation of A549/DDP cells was significantly decreased, and IC50 was significantly down-regulated; e CHAF1B substrate screening. *p < 0.05, ****p < 0.0001

To explore the mechanism of cisplatin resistance in lung adenocarcinoma, whole-genome chip screening was performed on A549/DDP and A549 cell lines. Comparing the two protein chips, a total of 7475 differential proteins were identified, and 5758 proteins were quantified. We defined proteins that were up-regulated than twice or down-regulated more than twice than A549 cells in A549/DDP cells as significant change proteins. There were 657 significantly changed proteins in the chip, of which 312 were up-regulated significantly and 345 were down-regulated significantly. There were 46 ubiquitinating enzymes in the up-regulated proteins, of which 42 were ubiquitin ligases (Fig. 1a). E3s play an important role in recognizing substrates during ubiquitination and are related to cisplatin resistance in malignant tumors. To clarify the function of E3s and explore whether they can affect the survival of lung adenocarcinoma patients, we consulted the public database (http://gepia.cancer-pku.cn/) and found that in 42 E3s, E3s including ARPC1A, AURKA, CDC20, CDCA3, CHAF1B, FBXO22, PPP1R13L and TRIP12 were negatively correlated with the prognosis of LUAD patients (Fig. 1b). Gepia suggests that the high expression of CHAF1B is negatively correlated with DFS of patients with lung adenocarcinoma. It is confirmed that the high expression of CHAF1B is negatively correlated with the prognosis of patients with lung adenocarcinoma in the public database Ualcan (http://ualcan.path.uab.edu/index.html) (Additional file 1: Figure S1).

CHAF1B, CDC20, PPP1R13L and TRIP12 were selected to explore, which was significantly up-regulated and negatively related to prognosis. The expression of CHAF1B, PPP1R13L and CDC20 in A549/DDP cells were up-regulated compared with A549 cells (*p *< 0.05), which was consistent with the trend of protein chip results (Fig. 1c). To further explore the effects of CHAF1B, PPP1R13L and CDC20 on the drug sensitivity of A549/DDP cells to cisplatin, construct the corresponding si-RNA knockdown of the three genes mentioned above in A549/DDP, then detect the proliferation of cancer cells by MTT method and calculate their IC50 and IC25. It was found that the proliferation of A549/DDP cells decreased significantly after knocking down the CHAF1B and PPP1R13L (Fig. 1d), and IC50 decreased significantly (Additional file 1: Table S1). Since PPP1R13L has been reported to be associated with cisplatin resistance and the IC50 decline after knocking down CDC20 was relatively small, this study explored the role of CHAF1B on cisplatin sensitivity in lung adenocarcinoma cells.

### Knockdown of CHAF1B in A549/DDP increased NCOR2 expression, while knockdown of NCOR2 decreased cisplatin sensitivity

**Fig. 2:**
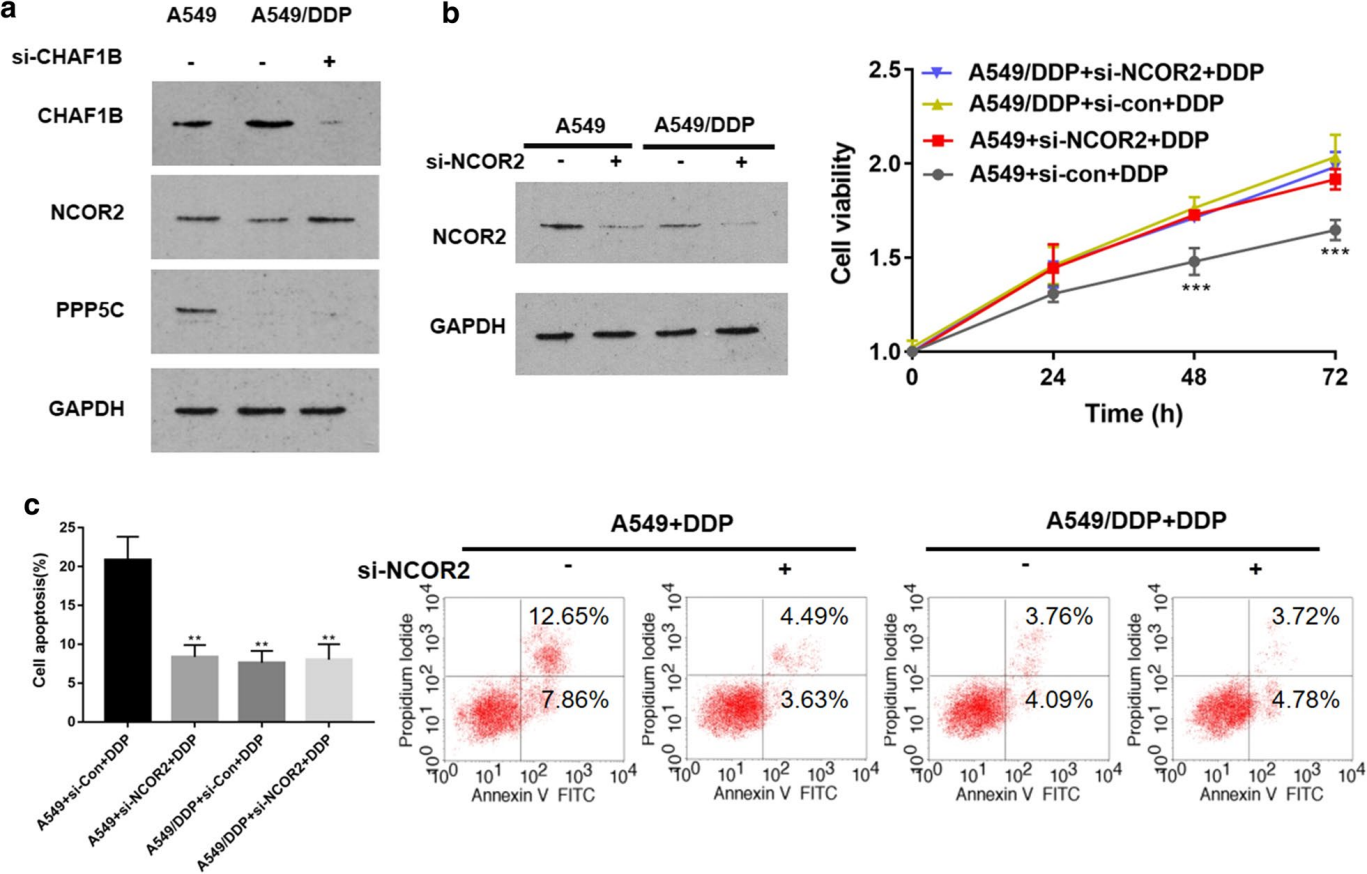
CHAF1B regulates NCOR2 expression and low NCOR2 expression leads to cisplatin resistance. **a** After CHAF1B was knocked down, NCOR2 expression increased while PPP5C did not change significantly; **b**, **c** MTT and flow cytometry showed that the sensitivity of lung adenocarcinoma cells to cisplatin decreased, cell proliferation increased and apoptosis decreased after NCOR2 was knocked down in A549 cells. **p < 0.01, ***p < 0.001. DDP: IC25 concentration of cisplatin to A549/DDP

Ubiquitin ligases play a role by acting on substrates. Combining proteomics chips and public databases IUUCD (http://iuucd.biocuckoo.org/), Phosphosite (https://www.photosite.org/Homeaction.action) and Ualcan, we predicted the possible substrates of CHAF1B. Only NCOR2 and PPP5C met the following requirements: it is down-regulated by more than 1.5 times in A549/DDP, interacts with CHAF1B, has a ubiquitination site and is lowly expressed in lung adenocarcinoma (Fig. 1e). The database photosite showed that NCOR2 and PPP5C contain multiple ubiquitination sites (Additional file 1: Table S2). After knocking down CHAF1B in A549/DDP by si-CHAF1B, the transfection efficiency of si-RNA was shown in Additional file 1: Figure S2 and the expression of CHAF1B, NCOR2 and PPP5C was detected by WB method. NCOR2 was up-regulated, while PPP5C was not significantly changed. WB results also confirmed that the expression of CHAF1B in A549/DDP was up-regulated compared with A549 (Fig. 2a).

In order to clarify the function of NCOR2, group A and group B took A549 as the research object and transfected si-con and si-NCOR2, respectively. Group C and D transfected si-con and si-NCOR2, respectively in A549/DDP. MTT and Flow cytometry assay results showed that the cell viability increased and apoptosis decreased after NCOR2 knockdown in A549, while that in A549/DDP transfected or not transfected si-NCOR2 showed high cell viability and low apoptosis, suggesting that the down-regulation of NCOR2 was one of the reasons for cisplatin resistance in LUAD cells (Fig. 2b, c).

**Fig. 3:**
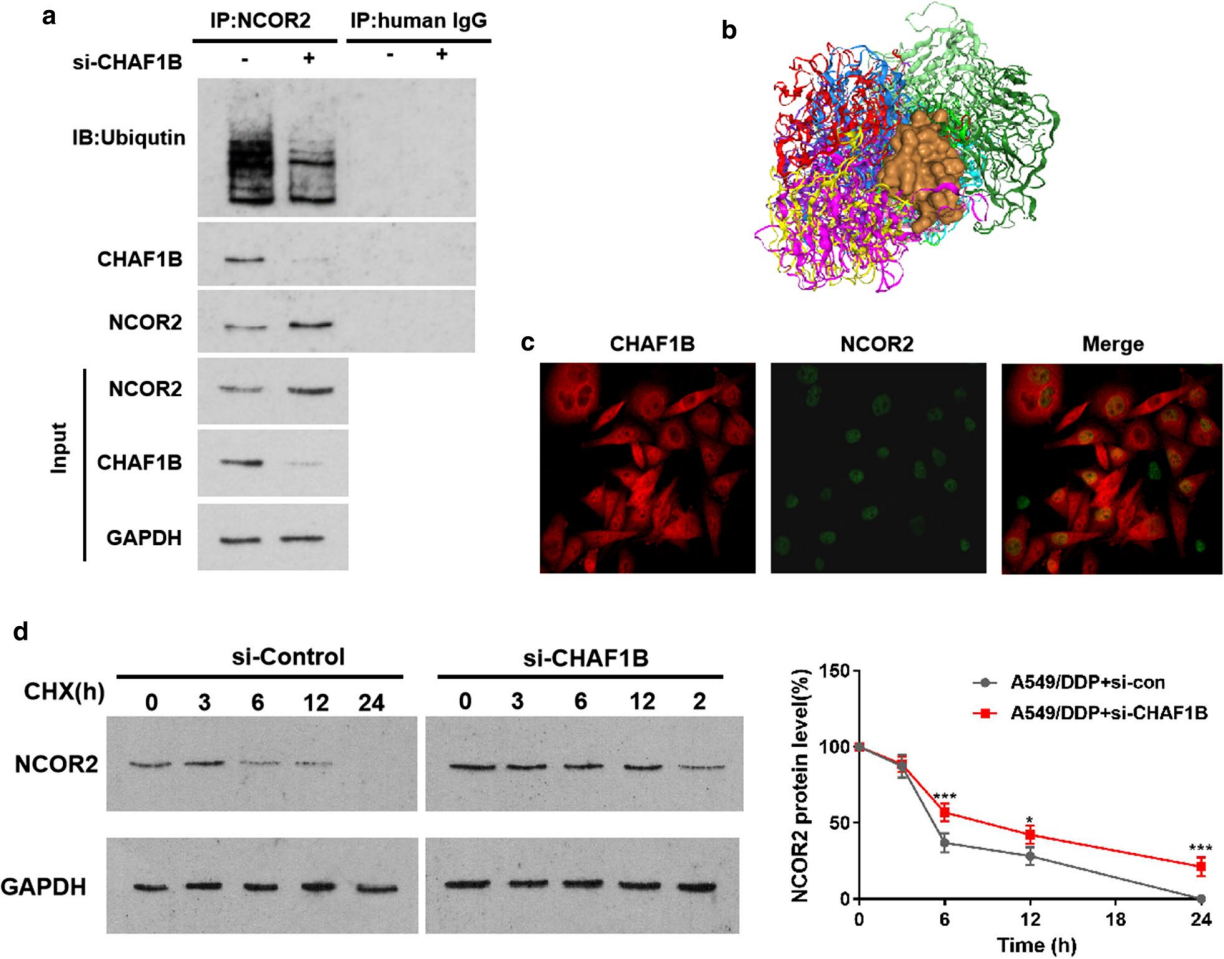
CHAF1B promote protein ubiquitination degradation of NCOR2. **a** Immunocoprecipitation showed that CHAF1B interacted with NCOR2. **b** A schematic diagram of the interaction between CHAF1B and NCOR2. Brown surface: CHAF1B, rainbow cartoon: NCOR2. **c** Immunofluorescence showed that NCOR2 (green) mainly existed in the nucleus, and CHAF1B (red) existed in both the nucleus and cytoplasm, both of which were co-located in the nucleus. **d** Protein stability test confirmed that NCOR2 degradation rate slowed down after CHAF1B was knocked down. *p < 0.05, ***p < 0.001

Immunocoprecipitation was used to explore the interaction between CHAF1B and NCOR2. The results showed that CHAF1B and NCOR2 were interacting proteins (Fig. 3a). HDOCK (http://hdock.phys.hust.edu.cn/) predicts the interaction pattern of CHAF1B and NCOR2 (Fig. 3b and Additional file 1: Figure S4), indicating that there are direct interaction between CHAF1B and NCOR2. Immunofluorescence (IF) assay showed that CHAF1B (red) existed in the nucleus and cytoplasm, and NCOR2 (green) in the nucleus, suggesting that the interaction between CHAF1B and NCOR2 was mainly located in the nucleus (Fig. 3c). The results of protein stability experiments indicated that the degradation rate of NCOR2 was significantly slowed down after knocking down CHAF1B (Fig. 3d). The above experiments confirmed that CHAF1B and NCOR2 are interacting proteins, and CHAF1B promotes NCOR2 ubiquitination degradation.

### Cell, animal experiments and clinical data confirmed that knockdown of CHAF1B could up-regulate NCOR2 and increase cisplatin sensitivity in LUAD

**Fig. 4:**
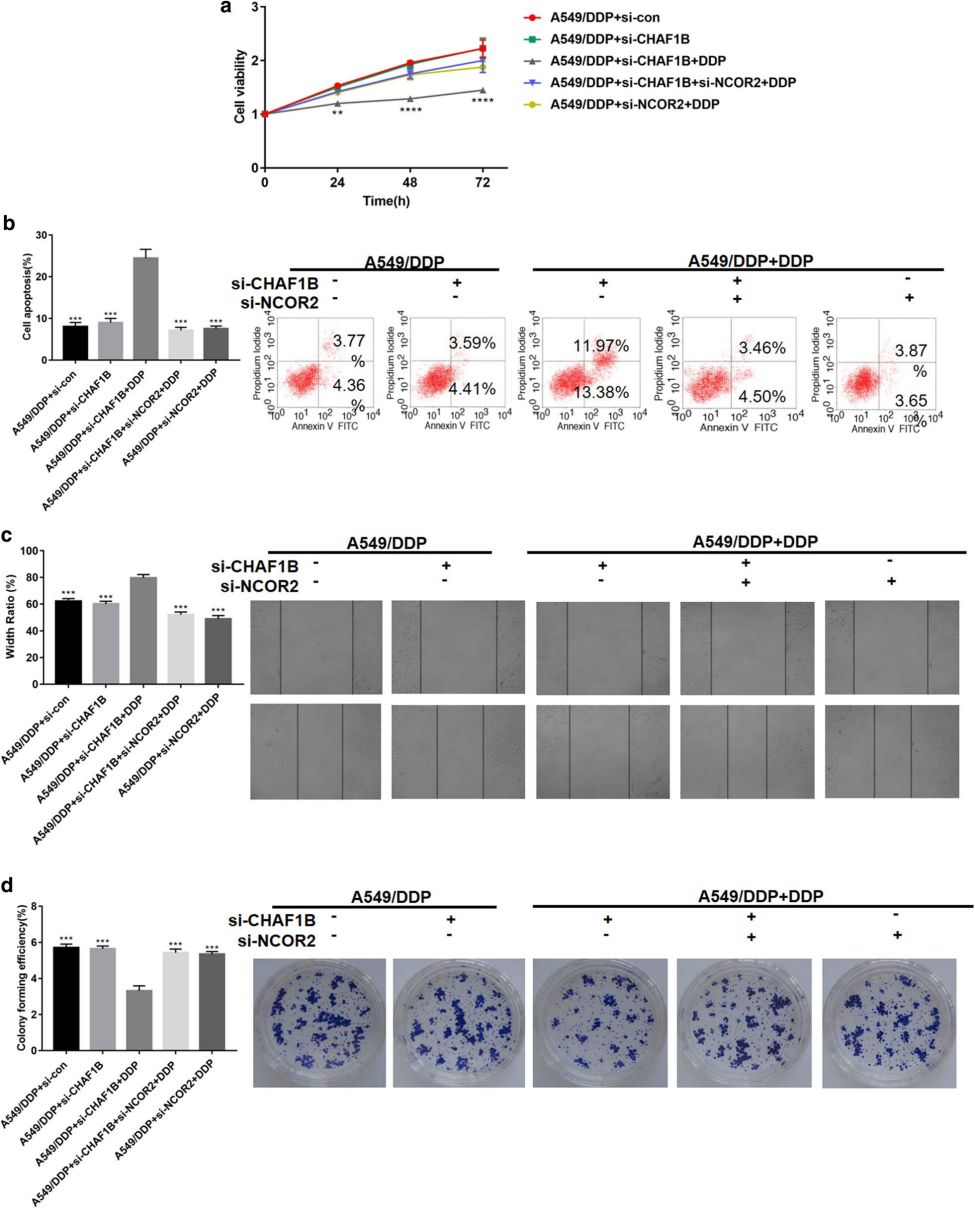
CHAF1B affects cisplatin sensitivity by regulating NCOR2. **a**–**d** MTT assay, flow cytometry, scratch assay and cell clone formation experiments were performed to verify that after knocking down CHAF1B, A549/DDP cells proliferation and metastasis decreased, and apoptosis increased. N = 3, **p < 0.01, ***p < 0.001, ****p < 0.0001

Functional recovery experiments were performed to explore the role of CHAF1B on cisplatin resistance by regulating NCOR2. A549/DDP was used as the research object to set up 5 groups of A to E. Groups A and B were transfected with si-control and si-CHAF1B respectively. Groups C and E were transfected with si-CHAF1B and si-NCOR2, respectively. Group D was simultaneously knocked down. CHAF1B and NCOR2, and the three groups of C to E were treated with DDP. After treatment, MTT assay (Fig. 4a), flow cytometry (Fig. 4b), scratch test (Fig. 4c) and cell clone formation test (Fig. 4d) were performed respectively. The results showed that after knocking down CHAF1B in A549/DDP, the cell proliferation ability decreased and apoptosis increased significantly. When CHAF1B and NCOR2 were knocked down, cell proliferation was restored and apoptosis decreased.

**Fig. 5:**
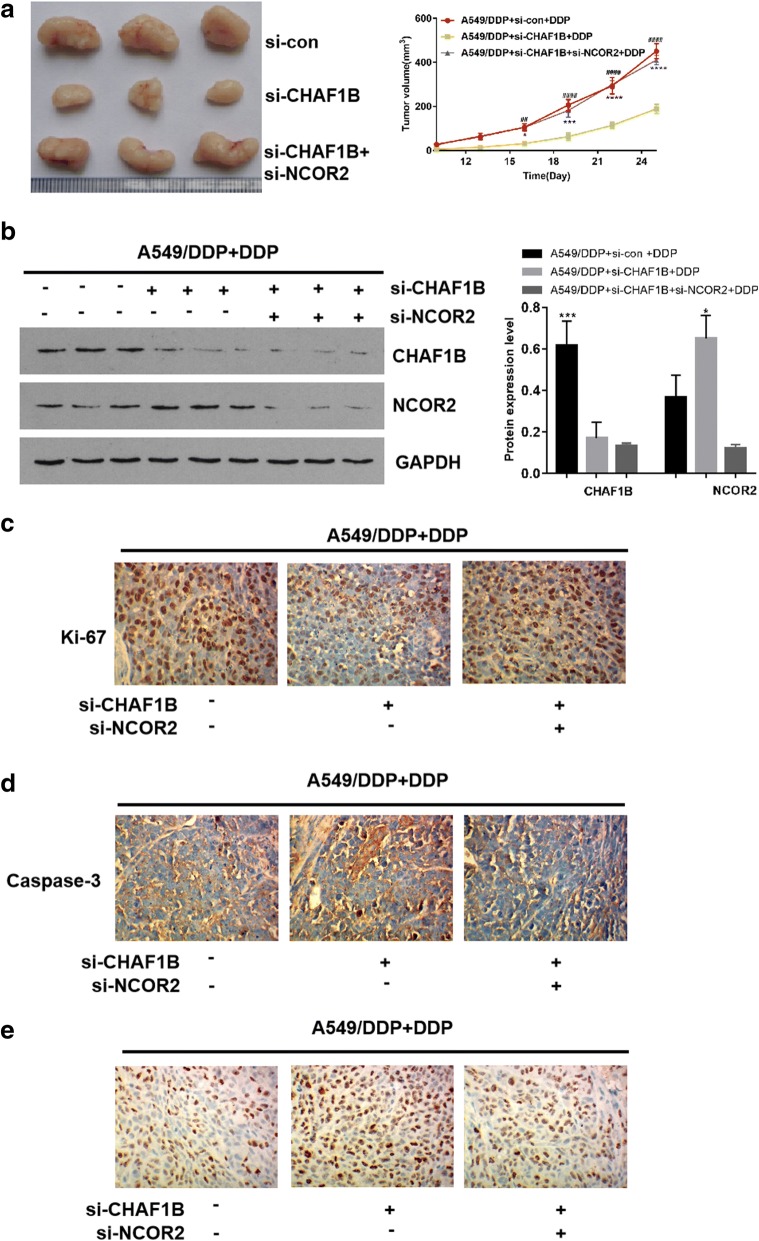
Animal experiments verify the function of CHAF1B and NCOR2. **a** The inhibition effect of cisplatin on the volume of transplanted tumors after A549/DDP knockdown of CHAF1B was significantly greater than that of the control group. **b** WB confirmed that the expression of CHAF1B was up-regulated in A549/DDP and NCOR2 was up-regulated in CHAF1B knockdown group. **c** The expression of Ki-67 was significantly lower in A549/DDP after knocking down CHAF1B than in the control group. **d** The TUNEL showed that apoptosis was significantly higher in A549/DDP after knocking down CHAF1B than that in the control group. Bars indicated standard deviation, *p < 0.05, ***p < 0.001, ****p < 0.0001. DDP: IC25 concentration of cisplatin to A549/DDP

**Fig. 6:**
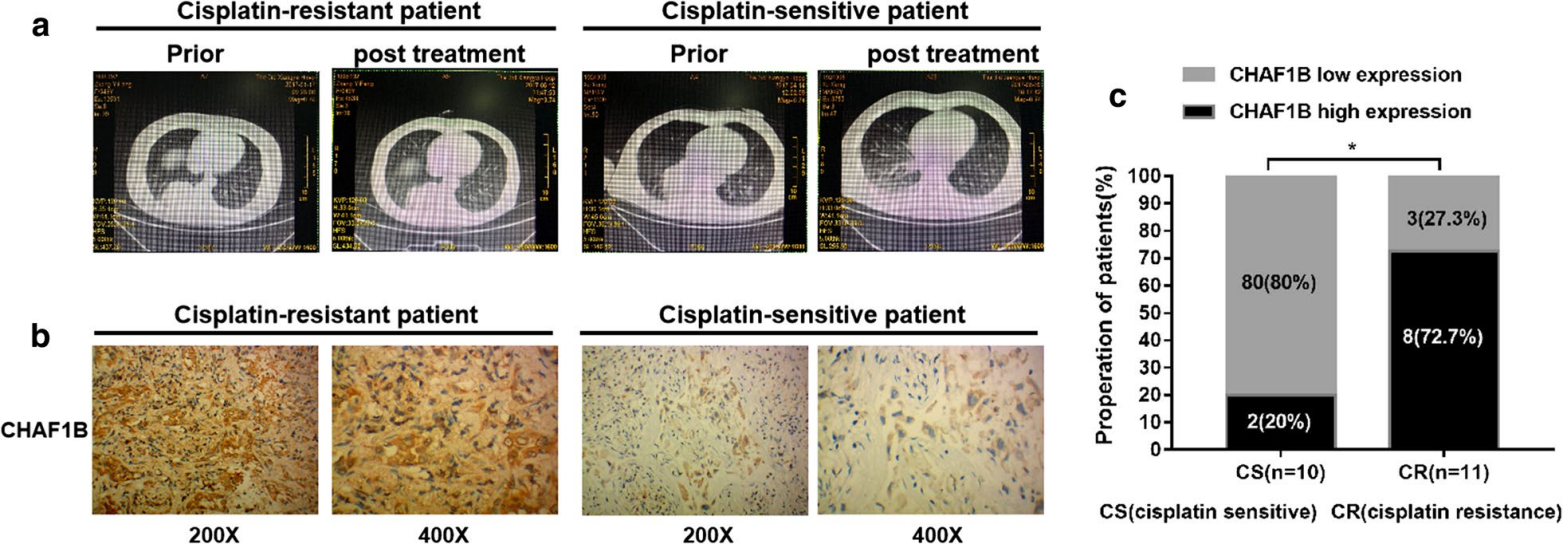
The CHAF1B expression in cisplatin-resistance LUAD tissues is higher than cisplatin-sensitive resistant tissues. **a**, **b** Measure CHAF1B expression in tissues LUAD tissues. Cisplatin-sensitive (n = 10) and cisplatin-resistant (n = 11) patients with CHAF1B expression are shown. With × 200 and × 400 times magnification under microscope, the CHAF1B expression in cisplatin-resistant patients were higher than that in cisplatin-sensitive patients. **c** Proportion of cisplatin-treated LUAD patients with low or high CHAF1B expression. *p < 0.05

In animal experiments, A549/DDP cells transfected with si-con, si-CHAF1B, and si-CHAF1B + si-NCOR2 respectively were transfected into mice for subcutaneous tumor transplantation. In CHAF1B knockdown group, the growth rate and size of the tumors treated with cisplatin were significantly lower than those of the control group (p < 0.05, Fig. 5a, b). WB confirmed that the CHAF1B expression was up-regulated in the tissues of A549/DDP group, and NCOR2 was up-regulated in the tumor tissues in CHAF1B knockdown group. By immunohistochemical detection of tumor proliferation-related index Ki-67, the positive rate of Ki67 in the CHAF1B knockdown group was significantly lower than that in the control group (Fig. 5c), and the apoptosis index was significantly up-regulated (Fig. 5d, e). Clinical CT and immunohistochemistry showed that CHAF1B expression was higher in cisplatin-resistant patients than that in cisplatin-sensitive patients (Fig. 6a–c). In summary, Vitro and Vivo experiments confirmed that CHAF1B promotes ubiquitination of NCOR2 and is one of the reasons of cisplatin resistance in LUAD patients.

## Discussion

Quantitative analysis of global proteome screened differentially expressed proteins between A549/DDP and A549. There were 46 ubiquitinating enzymes up-regulated proteins. The latest research shows that ubiquitin ligase has an important role in the recognition of substrates during ubiquitination. Therefore, we studied 42 E3s. E3, which was found to be negatively correlated with prognosis in public databases, included: ARPC1A, CHAF1B, TRIP12, CDC20, CDCA3, FBXO22, PPP1R13L, AURKA.

Some of the above E3s have been reported to be associated with cisplatin sensitivity. It has been reported that ubiquitin ligase FBXO22 [21] mediates the multiubiquitination and degradation of CD147 by interacting with the intracellular domain CD147-ICD of transmembrane glycoprotein CD147, thus reversing the cisplatin resistance of SMMC-7721, Huh-7 and A549 cancer cells. AURKA [22] is activated in gastric cancer and it has been demonstrated that AURKA promotes cisplatin resistance in gastric cancer by regulating eIF4E, c-MYC, HDM2. In this study, we selected the proteins that were not reported related to cisplatin resistance in literature for further study, including CDC20, CHAF1B, PPP1R13L, TRIP12. The PCR results showed that the expression levels of CHAF1B, PPP1R13L and CDC20 in A549/DDP cells were up-regulated compared with A549 cells. MTT showed that the proliferation of A549/DDP cells and IC50 decreased significantly after knockdown of the above three genes. In the research process, it has been reported that PPP1R13L [23] promotes EMT through the microRNA-20a-FBXL5/BTG3 signaling pathway and endows cervical cancer cells with cisplatin resistance. In this study, we investigated the regulatory mechanism of CHAF1B on cisplatin sensitivity in lung adenocarcinoma, because its effect on IC50 is greater than that of CDC20.

Combined with public database, PCR, WB and protein chip results, we know that CHAF1B is higher in cancer tissues than in adjacent tissues, and CAHF1B is the highest in resistant strains. Chromatin assembly factor 1 subunit B (CHAF1B) is proved to assembles histone octamers onto replicating DNA and mediate DNA repair [24, 25]. It has been confirmed that CHAF1B is overexpressed in laryngeal cancer, squamous cell carcinoma [26] ,high-grade gliomas,prostatic cancer and lung cancer, and its high expression leads to a shorter survival period for high-grade gliomas [27] ,prostatic cancer [28] and patients lung cancer. Cellular experiments confirmed that knockdown of CHAF1B inhibits the proliferation and invasion of cancer cells and inhibits the growth of xenografts in vitro [29–31]. Recent studies show that CHAF1B acts as a novel marker of tumor proliferation and prognosis. The literature suggest that ubiquitin ligase can affect the cisplatin drug sensitivity of malignant tumor by regulating the substrate expression. We referred to the public database IUUCD and found that CHAF1B is a ubiquitin ligase, which belongs to the category of cullin ring (Additional file 1: Figure S3).

Combining proteome chip, protein interaction website Biocuckoo, ubiquitination site prediction website Phosphosite and gene expression database Ualcan, we speculate that NCOR2 and PPP5C may interact with CHAF1B. WB results indicated that NCOR2 was up-regulated after CHAF1B was knocked down. MTT results showed that the cell viability increased and apoptosis decreased after NCOR2 was knocked down in A549 cell line, which suggested that the decrease of NCOR2 may be the cause of cisplatin resistance in lung adenocarcinoma.

Nuclear receptor corepressor 2 (NCOR2) is a member of the family of co-inhibitors associated with thyroid hormones and retinoic acid receptors. This protein acts as part of a multi-subunit complex of histone deacetylase to modify the chromatin structure and inhibits basal transcriptional activity of the target gene [32]. Abnormal expression of NCOR2 is associated with certain cancers. High expression of heterochromatin 1γ is associated with poor prognosis of lung adenocarcinoma and promotes proliferation, colony formation and migration of lung adenocarcinoma cells by directly inhibiting NCOR2 expression [33]. Studies have shown that down-regulation of NCOR2 [34] enhances the invasive ability of breast cancer cells and increase the growth and metastasis of breast cancer tumors in nude mice. In conclusion, NCOR2 may be an important inhibitor of cancer occurrence or development. Combined with the protein chip and experimental results, it was confirmed that the low expression of NCOR2 in lung adenocarcinoma cells may be one of the reasons for cisplatin resistance in lung adenocarcinoma.

Looking up database photosite, we found that NCOR2 contains multiple ubiquitination sites. HDOCK was used to predict the role and docking site of CHAF1B and NCOR2, indicating that there are direct interactions between CHAF1B and NCOR2. The CO-IP, IF and protein stability experiments results showed that CHAF1B and NCOR2 were interacting proteins in the nucleus and CHAF1B promoted NCOR2 degradation. Bioinformatics Analysis, cellular and animal experiments have demonstrated that CHAF1B can cause cisplatin resistance by increasing ubiquitination degradation of NCOR2.

Therefore, our results support that ubiquitin ligase induces cisplatin resistance in lung adenocarcinoma by promoting substrate degradation, and ubiquitin ligases CHAF1B and NCOR2 can be considered as potential biomarkers for cisplatin sensitivity in lung adenocarcinoma. At present, cell, animal and clinical experiments have been completed in this study. In the future, we will explore the specific ubiquitination sites and types of CHAF1B regulating NCOR2. The relationship between ubiquitin ligase and cisplatin sensitivity may provide a new way to reverse drug resistance of lung adenocarcinoma and promote the development of effective treatment strategies.

## Conclusion

Ubiquitin ligase CHAF1B leads to cisplatin resistance in lung adenocarcinoma cells by promoting NCOR2 ubiquitination degradation.

## Supplementary information

**Additional file 1.** Additional figures and tables.

## Data Availability

All data generated or analysed during this study are included in this published article and its additional file.

## Abbreviations

E1: Ubiquitin activating enzyme
E2: Ubiquitin conjugating enzyme
E3: Ubiquitin ligase
CHAF1B: Chromatin assembly factor 1 subunit B
NCOR2: Nuclear receptor corepressor 2
LUAD: Lung adenocarcinoma
CO-IP: CO-immunocoprecipitation
IHC: Immunohistochemical
qRT-PCR: Quantitative real-time quantitative polymerase chain reaction
siRNA: Small interfering RNA
IF: Immunofluorescence
