# Supplementary material for: Ubiquitin ligase CHAF1B induces cisplatin resistance in lung adenocarcinoma by promoting NCOR2 degradation
Source: Cancer Cell Int. 2020 May 25;20:194. doi: 10.1186/s12935-020-01263-2 (PMC7249347; doi:10.1186/s12935-020-01263-2)
Supplement: Supplementary file 1 — Additional file 1. Additional figures and tables. [file 12935_2020_1263_MOESM1_ESM.docx]

Additional file


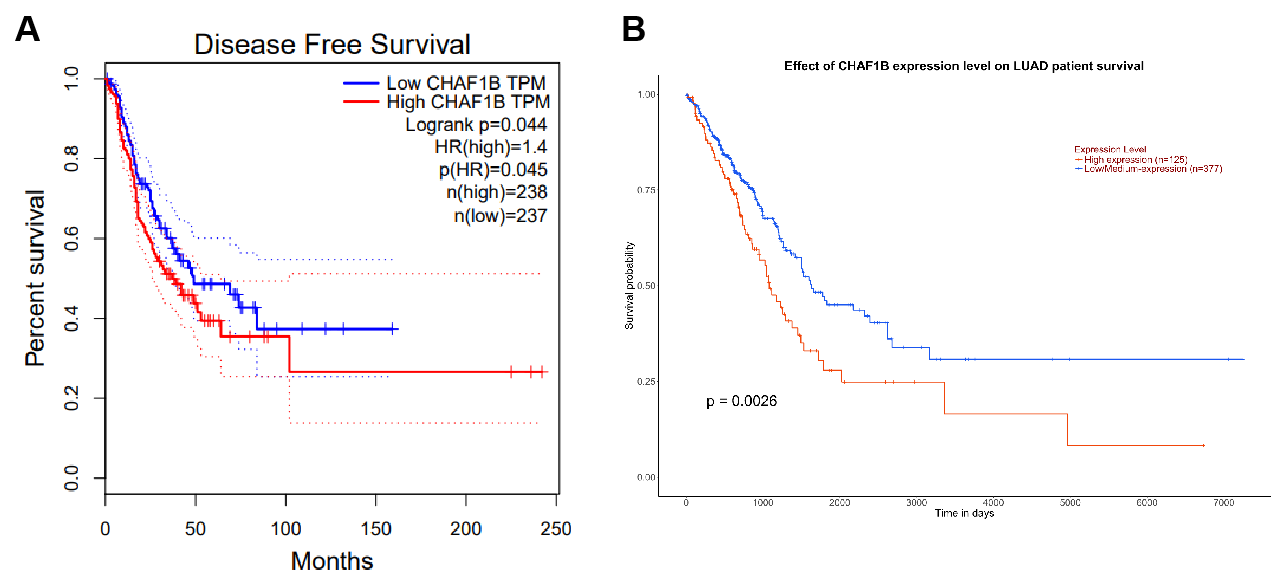


**Additional file 1: Figure S1**

A, B E3 CHAF1B was negatively correlated with the prognosis of patients with lung adenocarcinoma.


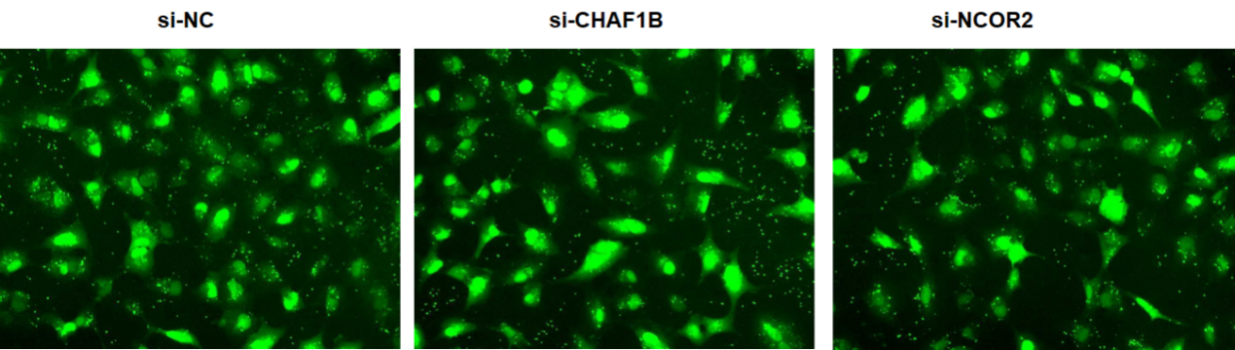


**Additional file 1: Figure S2**

Fluorescence microscopy indicating siRNA transfected A549 cells successfully


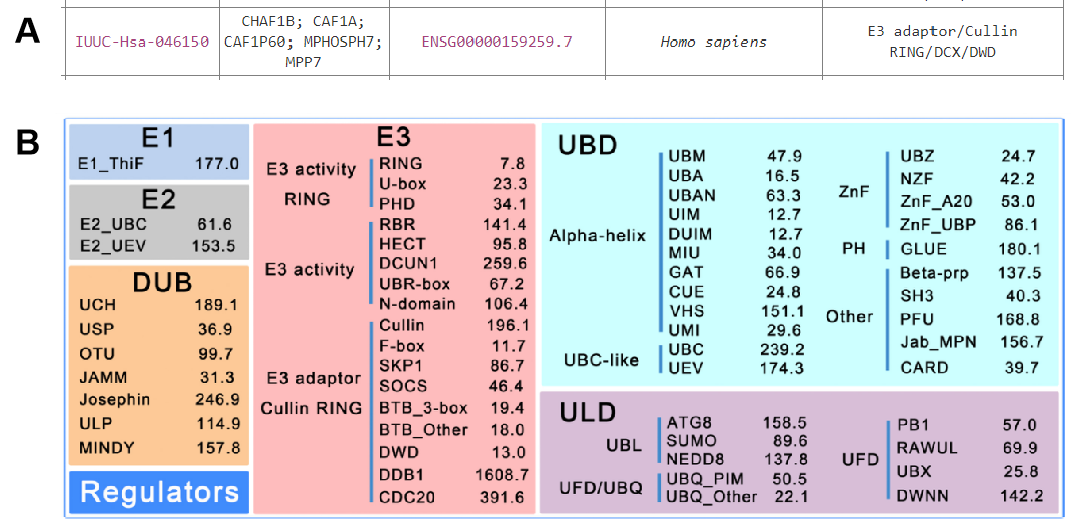


**Additional file 1: Figure S3**

A IUUCD suggests CHAF1B is E3. B IUUCD suggests the classification of ubiquitinase.

**
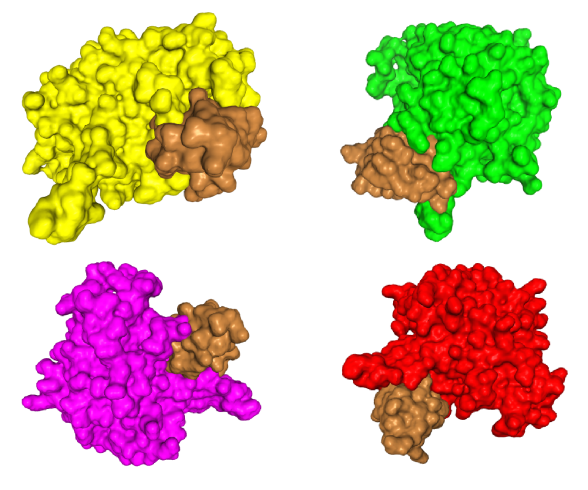

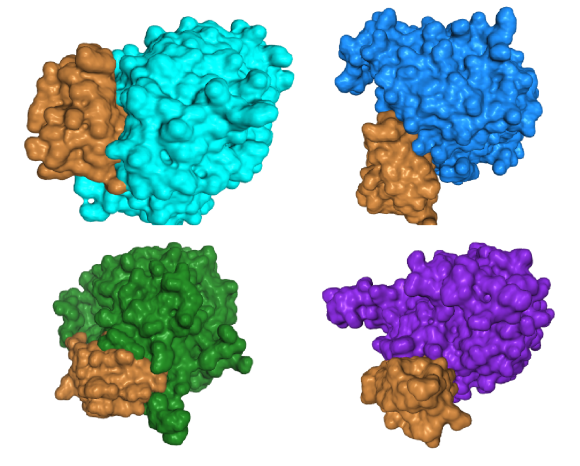

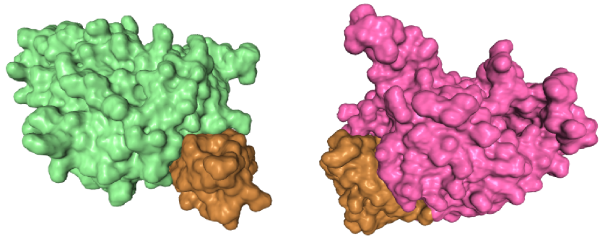
**

**Additional file 1: Figure S4**

HDOCK predicting the role and docking site of CHAF1B and NCOR2, indicating that there are direct interactions between CHAF1B and NCOR2. Brown surface: CHAF1B, other colored surface:NCOR2.

**Additional file 1: Table S1. 95% confidence interval of IC50**

|  | A549  +si-con | A549/DDP  +si-con | A549/DDP  +si-CHAF1B | A549/DDP  +si-CDC20 | A549/DDP  +si-PPP1R13L |
| --- | --- | --- | --- | --- | --- |
| ug/ml | 8.42 to 9.28 | 49.97 to 63.70 | 15.91 to 18.95 | 23.85 to 39.66 | 26.92 to 35.59 |

**Additional file 1: Table S2. Prediction of NCOR2 ubiquitination sites**

| [K106-ub](https://www.phosphosite.org/siteAction.action?id=964546125) | EMEFIESkRPRLELL |
| --- | --- |
| [K137-ub](https://www.phosphosite.org/siteAction.action?id=41495671) | AGSEDLTkDRSLTGK |
| [K271-ub](https://www.phosphosite.org/siteAction.action?id=964546145) | RQYHENIkINQAMRK |
| [K1160-ub](https://www.phosphosite.org/siteAction.action?id=964546127) | PLPMDPKkLAPFSGV |
| [K1210-ub](https://www.phosphosite.org/siteAction.action?id=41495679) | VPGGsITkGIPSTRV |
| [K1273-ub](https://www.phosphosite.org/siteAction.action?id=964546129) | GHVIyEGkKGHVLSY |
| [K1308-ub](https://www.phosphosite.org/siteAction.action?id=964546131) | PHEtAAPkRTYDMME |
| [K1408-ub](https://www.phosphosite.org/siteAction.action?id=964546133) | ALGPLKLkPAHEGLV |
| [K1458-ub](https://www.phosphosite.org/siteAction.action?id=964546135) | ItQGtPLkYDTGAsT |
| [K1509-ub](https://www.phosphosite.org/siteAction.action?id=41495683) | ACyEESLkSRPGtAS |
| [K1578-ub](https://www.phosphosite.org/siteAction.action?id=41495687) | EGsLSSSkASQDRkL |
| [K2026-ub](https://www.phosphosite.org/siteAction.action?id=964546137) | HREkTQSkPFSIQEL |
| [K2069-ub](https://www.phosphosite.org/siteAction.action?id=964546139) | tHDKGLPkHLEELDk |
| [K2076-ub](https://www.phosphosite.org/siteAction.action?id=964546141) | kHLEELDksHLEGEL |
| [K2086-ub](https://www.phosphosite.org/siteAction.action?id=964546143) | LEGELRPkQPGPVKL |

**Prediction of PPP5C ubiquitination sites**

| [K26-ub](https://www.phosphosite.org/siteAction.action?id=573765557) | PPADGALkRAEELkt |
| --- | --- |
| [K32-ub](https://www.phosphosite.org/siteAction.action?id=21182249) | LkRAEELktQANDYF |
| [K40-ub](https://www.phosphosite.org/siteAction.action?id=21182257) | tQANDYFkAkDYENA |
| [K42-ub](https://www.phosphosite.org/siteAction.action?id=573765559) | ANDYFkAkDYENAIk |
| [K49-ub](https://www.phosphosite.org/siteAction.action?id=21182251) | kDYENAIkFYSQAIE |
| [K97-ub](https://www.phosphosite.org/siteAction.action?id=573765561) | ELDkKYIkGYYRRAA |
| [K111-ub](https://www.phosphosite.org/siteAction.action?id=573765563) | AsNMALGkFRAALRD |
| [K124-ub](https://www.phosphosite.org/siteAction.action?id=964537505) | RDyEtVVkVKPHDKD |
| [K185-ub](https://www.phosphosite.org/siteAction.action?id=21182255) | GPKLEDGkVTISFMK |
| [K199-ub](https://www.phosphosite.org/siteAction.action?id=41493186) | KELMQWYkDQKKLHR |
| [K232-ub](https://www.phosphosite.org/siteAction.action?id=964537507) | TLVETTLkETEKITV |
| [K320-ub](https://www.phosphosite.org/siteAction.action?id=21182253) | yGFEGEVkAKYTAQM |
| [K412-ub](https://www.phosphosite.org/siteAction.action?id=573765567) | QFGPDVTkAFLEENN |
| [K430-ub](https://www.phosphosite.org/siteAction.action?id=573765569) | IIRSHEVkAEGyEVA |
